# The Involvement of miR-23a/*APAF1* Regulation Axis in Colorectal Cancer

**DOI:** 10.3390/ijms150711713

**Published:** 2014-07-02

**Authors:** Fung Lin Yong, Chee Woon Wang, April Camilla Roslani, Chee Wei Law

**Affiliations:** 1 Department of Surgery, Faculty of Medicine, University of Malaya, Kuala Lumpur 50603, Malaysia; E-Mails: aprilroslani@um.edu.my (A.C.R.); drcwlaw@gmail.com (C.W.L.); 2 Department of Biochemistry, Faculty of Medicine, MAHSA University, Kuala Lumpur 59100, Malaysia; E-Mail: wang.chee@mahsa.edu.my

**Keywords:** miR-23a, APAF1, colorectal cancer, apoptosis, SW480, SW620

## Abstract

Recent advances in microRNAome have made microRNAs (miRNAs) a compelling novel class of biomarker in cancer biology. In the present study, the role of miR-23a in the carcinogenesis of colorectal cancer (CRC) was investigated. Cell viability, apoptosis, and caspase 3/7 activation analyses were conducted to determine the potentiality of apoptosis resistance function of miR-23a in CRC. Luciferase assay was performed to verify a putative target site of miR-23a in the 3'-UTR of apoptosis protease activating factor 1 (*APAF1*) mRNA. The expression levels of miR-23a and *APAF1* in CRC cell lines (SW480 and SW620) and clinical samples were assessed using reverse transcription-quantitative real-time PCR (RT-qPCR) and Western blot. We found that the inhibition of miR-23a in SW480 and SW620 cell lines resulted in significant reduction of cell viability and promotion of cell apoptosis. Moreover, miR-23a up-regulation was coupled with *APAF1* down-regulation in CRC tissue samples. Taken together, miR-23a was identified to regulate apoptosis in CRC. Our study highlights the potential application of miR-23a/*APAF1* regulation axis in miRNA-based therapy and prognostication.

## 1. Introduction

Colorectal cancer (CRC) is the third most common cancer worldwide [1]. The carcinogenesis of CRC is heterogeneous, multi-factorial, and may take several decades. CRC is a curable disease if the growth is detected at an early stage. The limitations of the currently available tools for CRC screening and surveillance have highlighted the necessity of finding novel biomarkers. Recently, microRNA (miRNA)-based studies have rendered remarkable contributions in the elucidation of carcinogenesis. miRNAs are small non-coding RNA molecules of ~22 nucleotides that act as regulators of gene expression through sequence complementarity between the miRNAs and the 3'-untranslated region (3'-UTR) of target mRNAs [2]. In the event of full complementarity, the target mRNA will be subjected to mRNA cleavage whereas partial complementarity will lead to translational repression and/or deadenylation whereby the mRNA will be localized to processing body (P-body) for storage or degradation [3,4,5,6].

miRNA deregulation has been observed in various cancer signaling pathways, including apoptosis. Apoptosis, also known as programmed cell death, is an important regulator of tissue homeostasis. The two common apoptosis pathways are extrinsic (death receptor-mediated) and intrinsic (mitochondrial) pathways [7]. miRNA deregulation in apoptosis contributes to CRC development and resistance to anti-cancer therapy. For instance, over-expression of miR-92a has been reported to cause uncontrollable cell proliferation in CRC via the down-regulation of pro-apoptotic molecule BCL-2-interacting mediator of cell death (Bim) [8]. Programmed cell death 4 (PDCD4), a tumor suppressor protein that is often down-regulated in CRC, is caused by the up-regulation of miR-21 [9,10]. Moreover, Chang *et al.* has revealed the significance of miR-34a in regulating p53 network of cell cycle control and apoptosis in CRC [11].

Previously through miRNA microarray study, we had demonstrated that miR-23a may serve as a potential biomarker for the detection of CRC [12]. The next logical move is to determine the functional roles and targets of miR-23a. A single miRNA is predicted to have multiple regulatory functions and targets in a particular cancer or disease [13]. Thus, the focus of this study is to investigate the potentiality of apoptosis resistance function of miR-23a in CRC.

## 2. Results and Discussion

### 2.1. Results

#### 2.1.1. miR-23a Up-Regulation in Clinical CRC Samples

**Figure 1:**
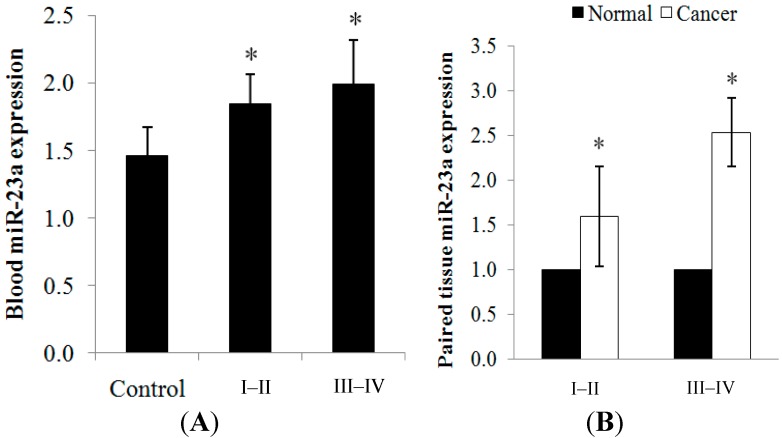
(**A**) miR-23a expression was detected in the blood samples from healthy controls and CRC patients. An increasing trend of expression was observed as the cancer progressed from stage I–II to stage III–IV tumors; and (**B**) miR-23a expression was detected in the paired cancer tissues. Relative expression is expressed as fold change of cancer tissue *versus* adjacent normal mucosa. RNU48 was used as the endogenous control in the RT-qPCR analysis. Fold change above 1 indicates up-regulation. Data are presented as mean ± standard error of mean (SEM). * *p* < 0.05.

The expression of miR-23a in CRC was evaluated using 102 blood samples (32 healthy controls; 70 patients) and 30 paired cancer tissues via RT-qPCR (Table 1). The clinical samples were categorized into less advanced tumor (stage I–II) and more advanced tumor (stage III–IV). miR-23a was significantly over-expressed in the blood and cancer tissues when compared to the control/normal counterparts. Positive correlation was detected between the blood and tissue miR-23a expression, with *r* = 0.827 (*p* < 0.01). An increasing trend of expression was observed as the tumor progressed from stage I–II (1.84 ± 0.23-fold in blood; 1.59 ± 0.56-fold in tissue) to stage III–IV (1.99 ± 0.33-fold in blood; 2.53 ± 0.38-fold in tissue) (Figure 1A,B). The expression level of each sample is tabulated in Supplementary Table S1.

**Table 1:** Clinical data of healthy controls and colorectal cancer (CRC) patients.

Characteristics		Healthy Control (*n* = 32), *n* (%)	CRC Blood (*n* = 70), *n* (%)	Subset of Paired CRC Tissue (*n* = 30), *n* (%)
Age (years)		58.0 ± 11.1	65.1 ± 9.6	65.2 ± 9.8
Gender	Male	19 (59.4%)	54 (77.1%)	19 (63.3%)
	Female	13 (40.6%)	16 (22.9%)	11 (36.7%)
Ethnicity	Asian	32 (100%)	70 (100.0%)	30 (100%)
TNM stage	I–II		39 (55.7%)	10 (33.3%)
	III–IV		31 (44.3%)	20 (66.7%)
Tumor location	Colon		42 (60.0%)	30 (100%)
	Rectum		28 (40.0%)	0
Tumor grading (adenocarcinoma)	G1		25 (35.7%)	13 (43.3%)
	G2		34 (48.6%)	13 (43.3%)
	G3		11 (15.7%)	4 (13.3%)

#### 2.1.2. Inhibition of miR-23a in CRC Cell Lines Reduces Cell Viability, Promotes Cell Apoptosis and Increases Caspase Activation

**Figure 2:**
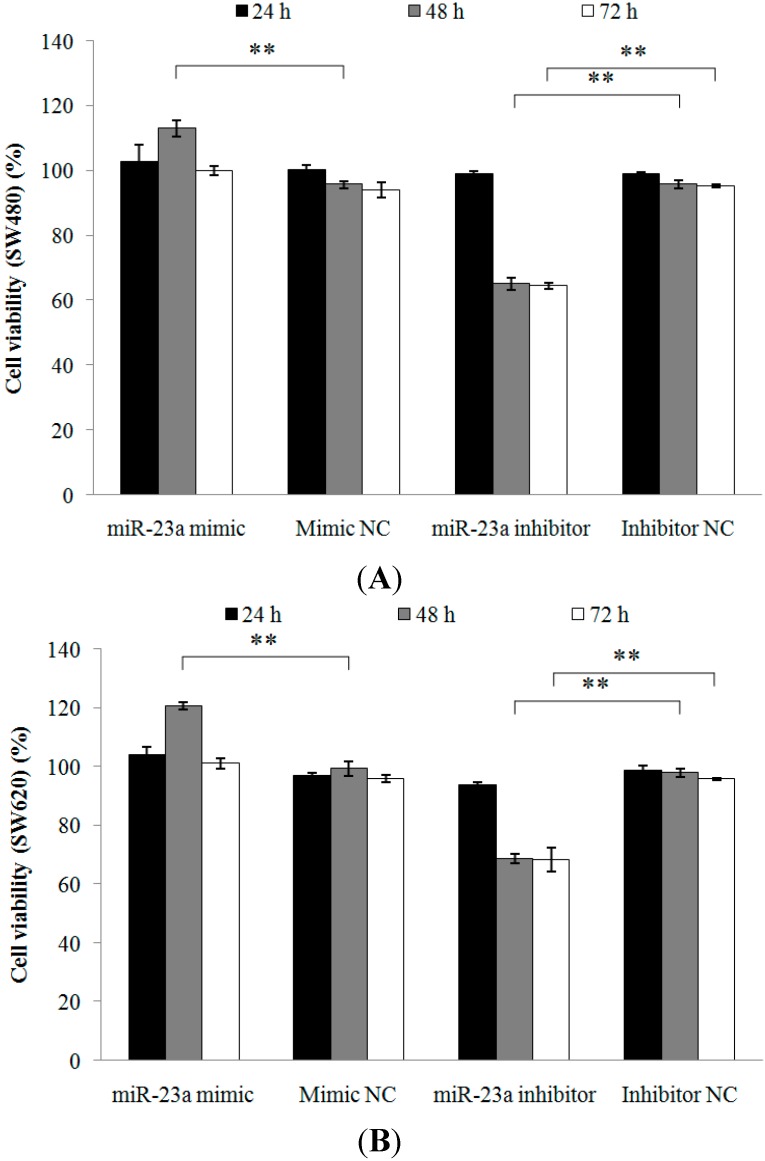
Cell viability of (**A**) SW480 and (**B**) SW620 cells following miR-23a mimic, miR-23a inhibitor or negative controls transfections. The cell viability was measured post transfections at 24, 48, and 72 h using 1× 3-(4,5-dimethylthiazol-2-yl)-2,5,-diphenyltetrazolium bromide (MTT) assay. miR-23a inhibitor transfection has successfully reduced cell viability at 48 and 72 h. Data are presented as mean ± SEM (*n* = 3). ** *p* < 0.01.

Two CRC cell lines with different metastatic potential (SW480 and SW620) were used to investigate the possibility of miR-23a-mediated apoptosis resistance effect in CRC. The SW480 cell line was derived from a primary Dukes’ type B tumor obtained from the colon of a 50-year-old male Caucasian whereas SW620 cell line was cultured a year later from a lymph node metastasis in the same patient diagnosed with Dukes’ type C tumor [14]. Cell viability, apoptosis, and caspase 3/7 activation analyses were carried out. Normalized data are expressed as relative value against the respective negative controls. The cell viability of SW480 and SW620 cells post transfections (24, 48 and 72 h) was measured. Cell viability of SW480 cells was significantly increased (118.08% ± 3.51%) and decreased (67.94% ± 1.77%) at 48 h following miR-23a mimic and miR-23a inhibitor transfections, respectively (Figure 2A). Cell viability of SW620 cells was also significantly increased (121.46% ± 2.70%) and decreased (70.22% ± 2.56%) at 48 h following miR-23a mimic and miR-23a inhibitor transfections, respectively (Figure 2B). At 72 h, only miR-23a inhibitor transfection revealed significant reduction of cell viability in both SW480 (67.85% ± 0.72%) and SW620 (71.24% ± 4.26%) cells. miR-23a mimic transfection has no effect on cell viability after 72 h. The cells may have entered the stationary phase due to prolong transfection, whereby cell proliferation is greatly reduced and further increase in cell viability is no longer possible. The optimal transfection period in the cell viability assay was 48 h.

**Figure 3:**
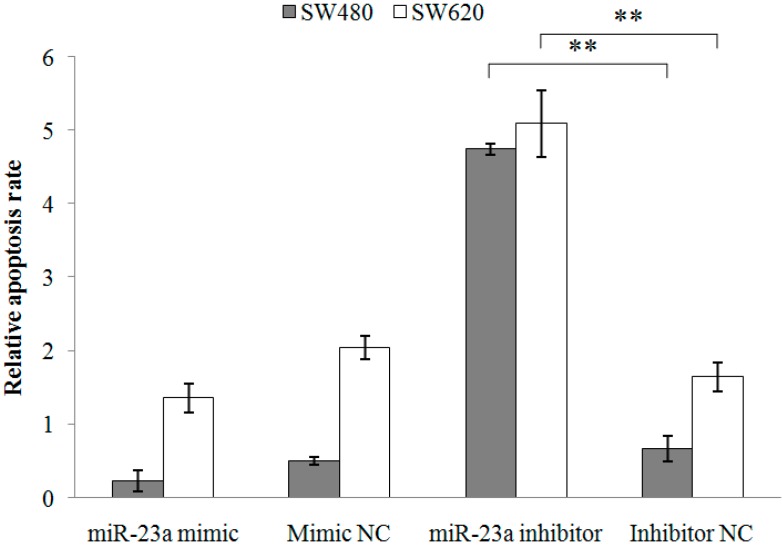
Relative apoptosis rate of SW480 and SW620 cells following miR-23a mimic, miR-23a inhibitor or negative controls transfections. The apoptosis rate was quantitated using Tali image-based cytometer. miR-23a inhibitor transfection has successfully elevated cell apoptosis. Fold change above 1 indicates up-regulation. Data are presented as mean ± SEM (*n* = 3). ** *p* < 0.01.

**Figure 4:**
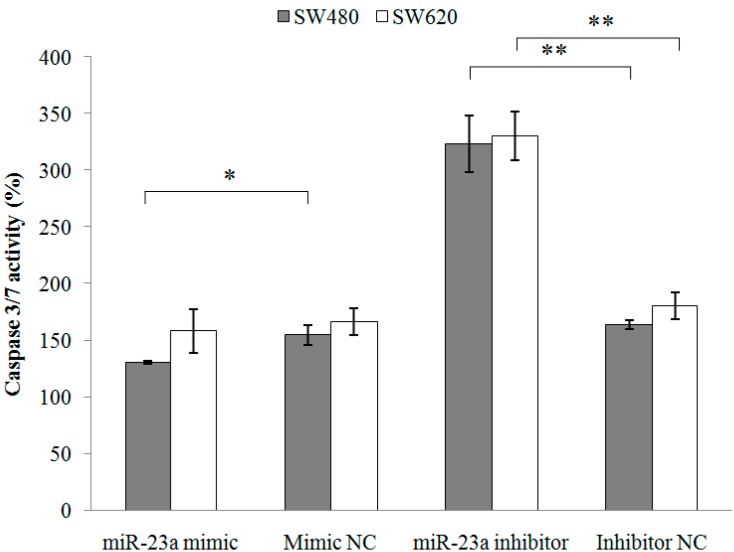
Caspase 3/7 activity of SW480 and SW620 cells following miR-23a mimic, miR-23a inhibitor or negative controls transfections. The caspase 3/7 activity was measured using Caspase-Glo 3/7 assay. miR-23a inhibitor transfection has successfully increased caspase 3/7 activity. Data are presented as mean ± SEM (*n* = 3). * *p* < 0.05, ** *p* < 0.01.

Next, apoptosis assay was conducted and the cell proportions in live, dead and apoptotic were quantitated using Tali image-based cytometer (Table 2). Doxorubicin, a well-known apoptosis-inducing agent, was used as the positive control. The cytometric analysis demonstrated low percentages of apoptotic cells in the SW480 and SW620 cells following miR-23a mimic transfection and high percentages of apoptotic cells following miR-23a inhibitor transfection. The relative apoptosis rate was then calculated and the transfection of miR-23a inhibitor resulted in a significantly higher proportion of apoptotic cells in both SW480 (8.11 ± 1.93-fold) and SW620 cells (3.24 ± 0.65-fold) when compared to the miRNA inhibitor negative control (Figure 3). Moreover, caspase 3/7 activation analysis was performed to further elucidate the apoptotic role of miR-23a in CRC. miR-23a inhibitor transfection induced significant activation of caspase 3/7 activity in both SW480 (198.42% ± 20.57%) and SW620 (183.27% ± 7.60%) cells (Figure 4). Collectively, the findings demonstrated that miR-23a inhibition reduced cell viability, promoted cell apoptosis, and increased caspase 3/7 activity in CRC cells.

**Table 2:** Quantification of live, dead and apoptotic cells in SW480 and SW620 cells using Tali image-based cytometer. Data are presented as mean ± SEM (*n* = 3).

Treatment	SW480			SW620		
	Live (%)	Dead (%)	Apoptotic (%)	Live (%)	Dead (%)	Apoptotic (%)
NTC	84.33 ± 0.33	10.33 ± 0.33	5.33 ± 0.33	84.00 ± 2.00	12.33 ± 1.76	3.67 ± 0.67
Mock	87.00 ± 1.53	11.00 ± 0.00	2.00 ± 1.53	81.67 ± 0.67	16.00 ± 0.58	2.00 ± 0.00
miR-23a mimic	85.33 ± 1.20	13.00 ± 1.00	1.33 ± 0.88	85.00 ± 1.15	10.00 ± 0.00	5.00 ± 1.15
Mimic NC	83.67 ± 0.88	13.00 ± 0.58	2.67 ± 0.33	78.33 ± 0.88	14.00 ± 0.58	7.33 ± 0.88
miR-23a inhibitor	65.67 ±1.45	9.00 ± 1.00	25.33 ± 1.86	68.00 ± 1.73	13.33 ± 0.67	18.33 ± 2.60
Inhibitor NC	85.00 ± 1.53	11.67 ± 0.67	3.67 ± 1.20	78.67 ± 1.20	15.00 ± 0.58	6.00 ± 1.15
Doxorubicin	54.33 ± 4.67	23.67 ± 0.88	22.33 ± 5.33	48.33 ± 4.84	27.00 ± 2.08	24.33 ± 2.91

#### 2.1.3. Functional Interaction between miR-23a and 3'-UTR of Apoptotic Peptidase Activating Factor 1 (*APAF1*) mRNA

**Figure 5:**
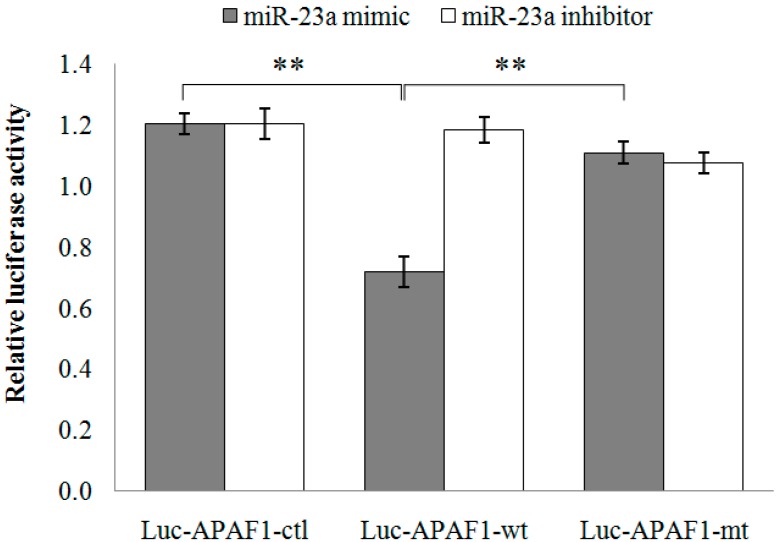
Relative luciferase activity following co-transfection of Luc-APAF1-ctl, Luc-APAF1-wt or Luc-APAF1-mt with miR-23a mimic or miR-23a inhibitor into SW480 cells. Luminescence values obtained from the transfected cells were background-subtracted with the non-transfected cells. Firefly/*Renilla* luciferase activity was expressed as relative value against the respective negative control. Functional interaction between Luc-APAF1-wt and miR-23a mimic was depicted via the down-regulation of luciferase activity with respect to Luc-APAF1-ctl. Restoration of luciferase activity was observed in the co-transfection of Luc-APAF1-mt and miR-23a mimic with respect to Luc-APAF1-wt. Fold change below 1 indicates down-regulation. Data are presented as mean ± SEM (*n* = 3). ** *p* < 0.01.

miRNA target prediction was computed using miRWalk database. The preliminary screening process yielded a long list of mRNAs. From there, *APAF1* was selected for further investigation, with the consideration of its role in apoptosis. At present, the common approach for miRNA-mRNA target validation is based on the combination of computational prediction and experimental validation. The binding of mature miRNA to its target is largely dependent on the free energy of binding between the seed region of miRNA and 3'-UTR of mRNA [15]. The predicted pairing region between miR-23a and *APAF1* mRNA is presented in Table 3. Two luciferase reporter constructs containing the wild-type *APAF1* 3'-UTR fragment (Luc-APAF1-wt) and the mutated *APAF1* 3'-UTR fragment (Luc-APAF1-mt) were utilized. Co-transfection of SW480 cells with Luc-APAF1-wt and miR-23a mimic has resulted in a significant down-regulation of luciferase activity (0.72 ± 0.05-fold) with respect to the luciferase vector without *APAF1* 3'-UTR (Luc-APAF1-ctl) while co-transfection with Luc-APAF1-mt and miR-23a mimic has resulted in a significant up-regulation of luciferase activity (1.11 ± 0.04-fold) when compared to the Luc-APAF1-wt (Figure 5). No significant differences were observed following co-transfections of Luc-APAF1-ctl, Luc-APAF1-wt or Luc-APAF1-mt with miR-23a inhibitor. From the results, mutation of the miR-23a target site in the *APAF1* 3'-UTR has successfully attenuated the miR-23a induced decrease of luciferase activity. Thus, miR-23a could influence the regulation of *APAF1* via the regulatory element present in the 3'-UTR region.

**Table 3:** Conserved miR-23a target site on human *APAF1* 3'-UTR (NM_013229.2). *APAF1* 3'-UTR length: 2836 bp. Seed match region was shown in bold italics. Mutations were marked with the symbol †.

APAF1 3'-UTR (nt 1474–1481)	5' ...AAGAUUUUUCUAAGAAAUGUGAA...
	| | | | | | |
hsa-miR-23a	3' CCUUUAGGGACCGUUACACUA
Mutated APAF1 3'-UTR (nt 1474−1481)	5' ...AAGAUUUUUCUAAGAAUUCUGCA...
	| † | † | | †
hsa-miR-23a	3' CCUUUAGGGACCGUUACACUA

#### 2.1.4. Modulation of *APAF1* Expression in SW480 and SW620 Cell Lines

**Figure 6:**
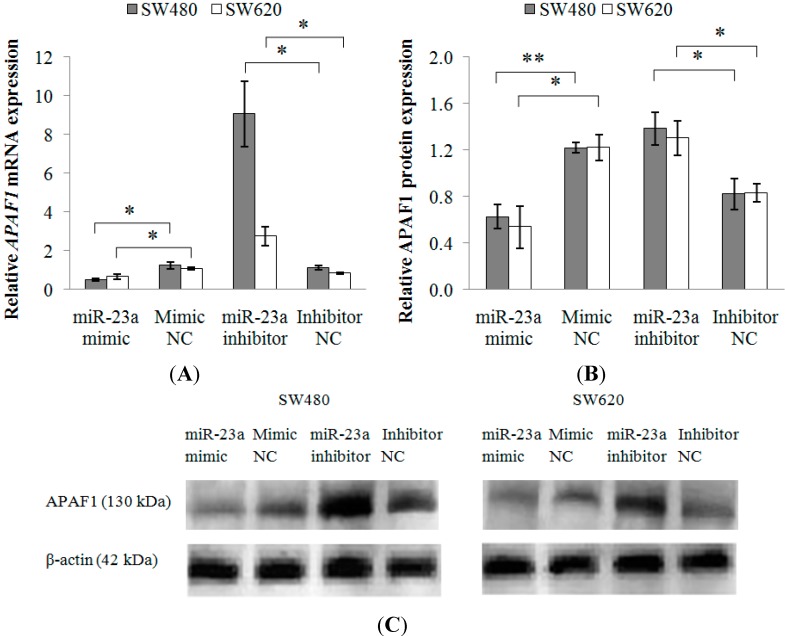
Relative *APAF1* mRNA and protein expressions in SW480 and SW620 cell lines following miR-23a mimic, miR-23a inhibitor or negative controls transfections. The expression levels of *APAF1* (**A**) mRNA and (**B**) protein were down-regulated in miR-23a mimic transfected cells and up-regulated in miR-23a inhibitor transfected cells. Fold change above 1 indicates up-regulation; and (**C**) the representative blots of APAF1 protein expression in SW480 and SW620 cells. β-actin was used as the loading control. Data are presented as mean ± SEM (*n* = 3). * *p* < 0.05, ** *p* < 0.01.

Following transfection with miR-23a mimic or miR-23a inhibitor (Table 4), monitoring of *APAF1* mRNA and protein expressions were conducted via RT-qPCR and Western blot, respectively. As depicted in Figure 6A, *APAF1* mRNA was significantly down-regulated in SW480 (0.43 ± 0.11-fold) and SW620 (0.62 ± 0.11-fold) cells transfected with miR-23a mimic and up-regulated in the SW480 (8.42 ± 1.99-fold) and SW620 (3.24 ± 0.49-fold) cells transfected with miR-23a inhibitor. Alterations in mRNA expression were reflected in protein expression as well. The Western blot analysis showed that the level of APAF1 protein was significantly decreased by 0.51 ± 0.08-fold in SW480 and 0.46 ± 0.17-fold in SW620 following miR-23a mimic transfection and significantly increased by 1.76 ± 0.28-fold in SW480 and 1.60 ± 0.22-fold in SW620 following miR-23a inhibitor transfection (Figure 6B,C). The transfection of miR-23a inhibitor has resulted in a relatively higher induction of *APAF1* mRNA and protein expressions in the non-metastatic SW480 cells when compared to the metastatic SW620 cells.

**Table 4:** Relative miR-23a expression in SW480 and SW620 cell lines following miR-23a mimic or miR-23a inhibitor transfection. Values are fold changes expressed as relative value against the respective negative controls. Data are presented as mean ± SEM (*n* = 3).

Treatment	Relative miR-23a Expression	
	SW480	SW620
miR-23a mimic	285.17 ± 50.11	234.59 ± 8.98
miR-23a inhibitor	0.03 ± 0.003	0.04 ± 0.005

#### 2.1.5. *APAF1* Down-Regulation in Clinical CRC Samples

**Figure 7:**
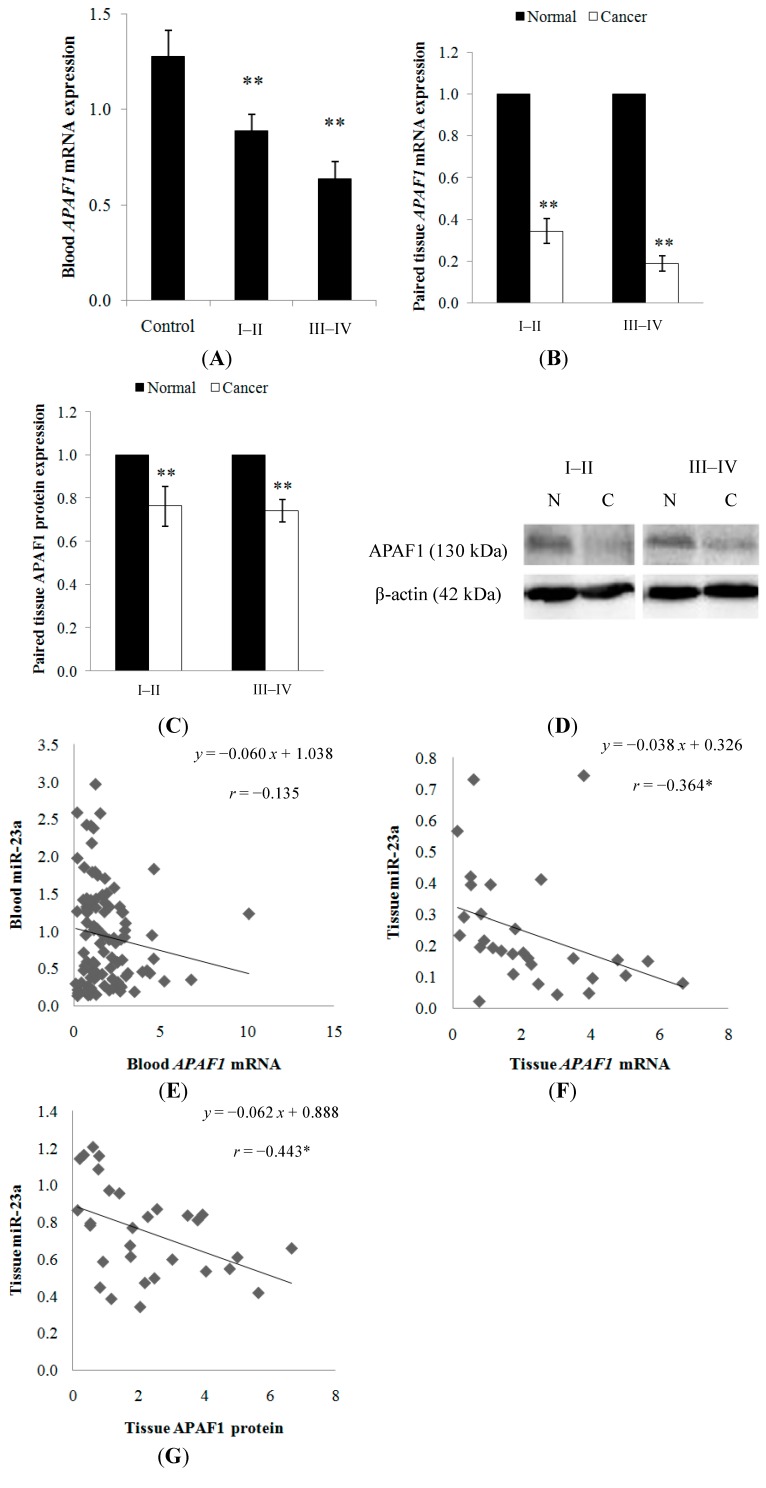
(**A**) *APAF1* expression was detected in the blood samples from healthy controls and CRC patients. A decreasing trend of expression was observed as the cancer progressed from stage I–II to stage III–IV tumors; (**B**) *APAF1* mRNA and (**C**) protein expressions were detected in the paired cancer tissues. Relative expression is expressed as fold change of cancer tissue *versus* the adjacent normal mucosa. Fold change below 1 indicates down-regulation; (**D**) the representative blots of APAF1 protein expression in the normal mucosa **N** and cancer tissue **C**. β-actin was used as the loading control; (**E**) correlation graph for blood miR-23a and *APAF1* mRNA; (**F**) correlation graph for tissue miR-23a and *APAF1* mRNA; and (**G**) correlation graph for tissue miR-23a and APAF1 protein. Data are presented as mean ± SEM. * *p* < 0.05, ** *p* < 0.01.

Similarly, the expression of *APAF1* was also evaluated using 102 blood samples (32 healthy controls; 70 patients) and 30 paired cancer tissues. *APAF1* mRNA expression was found to be significantly down-regulated in the clinical CRC samples. Pearson’s correlation between the blood and tissue *APAF1* level was 0.378 (*p* < 0.05). A decreasing trend of expression was observed as the tumor progressed from stage I–II (0.89 ± 0.09-fold in blood; 0.34 ± 0.06-fold in tissue) to stage III–IV (0.64 ± 0.09-fold in blood; 0.19 ± 0.04-fold in tissue) (Figure 7A,B). In addition, APAF1 protein expression was determined to be lower in the cancer tissue when compared to the adjacent normal mucosa, with 0.76 ± 0.09-fold in stage I–II and 0.74 ± 0.05-fold in stage III–IV (Figure 7C,D). The expression level of each sample is shown in Supplementary Table S1.

The relationship between miR-23a and *APAF1* mRNA or protein level were analyzed. Although no significant negative correlation was detected between miR-23a and *APAF1* mRNA (*r* = −0.135; *p* > 0.05) in the blood samples, miR-23a was found to be inversely correlated with *APAF1* mRNA (*r* = −0.364; *p* < 0.05) and protein (*r* = −0.443; *p* < 0.05) in the paired tissue samples (Figure 7E–G).

### 2.2. Discussion

In this study, miR-23a expression was determined to be up-regulated in the CRC blood and tissue samples. The elucidation of apoptosis function in the *in vitro* system via cell viability, apoptosis, and caspase 3/7 activation analyses revealed significant reduction of cell viability and promotion of cell apoptosis following miR-23a inhibitor transfection. *APAF1* mRNA was shown to be directly targeted by miR-23a through luciferase assay. Mutagenesis at the seed match region of *APAF1* mRNA has affirmed the specificity of miR-23a recognition. These findings supported our hypothesis whereby miR-23a could regulate apoptosis by targeting *APAF1*. Previously, Huerta *et al.* have reported that the endogenous *APAF1* expression was significantly lower in SW620 than SW480 cells [16]. The transfection of miR-23a inhibitor in the present study revealed relatively stronger induction of *APAF1* mRNA and protein expressions in the non-metastatic SW480 cells when compared to the metastatic SW620 cells. The SW620 cell line is believed to have lower susceptibility to gene modulation and apoptosis induction due to increased genetic mutations [16,17].

Independently, studies on miR-23a up-regulation and *APAF1* down-regulation in relation to apoptosis have been reported. For instance, Li *et al.* showed that miR-23a was involved in the regulation of cell death and apoptosis in mice thymic lymphoma model by targeting pro-apoptotic factor Fas [18]. The up-regulation of miR-23a has also been reported to down-regulate the expression of UVB-induced topoisomerase 1, caspase-7 and STK4 apoptotic factors in HaCaT cells [19]. On the other hand, *APAF1* down-regulation has been linked to decreased apoptosis and correlated with adverse prognosis in CRC [20,21]. In this study, significant inverse correlation between miR-23a and *APAF1* expression was detected in the tissue samples. Although statistical significance was not achieved in the negative correlation between blood miR-23a and *APAF1*, it is worthy of note that the complex environment of the circulatory system may hinder the absolute confirmation of whether the deregulated miR-23a and *APAF1* expressions are related to CRC alone or as a general mechanism in histological progression to cancer [22,23]. The detailed mechanism on the implication of cancer-derived miRNA in the circulatory system is yet to be elucidated. Nevertheless, as the tumor progressed from the less advanced stage (I–II) to the more advanced stage (III–IV), an increasing trend of miR-23a level and a decreasing trend of *APAF1* level were observed in both blood and tissue samples. A minute change in miRNA expression has been determined to have large-scale effects in fundamental cellular processes [24]. Moreover, a single miRNA is predicted to have multiple mRNA targets (around 200 transcripts) that work in concert in controlling a common pathway [13]. Hence, this study serves as a proof-of-concept example to highlight the feasibility of miR-23a/*APAF1* regulation axis as a biomarker to monitor tumor progression in CRC.

**Figure 8:**
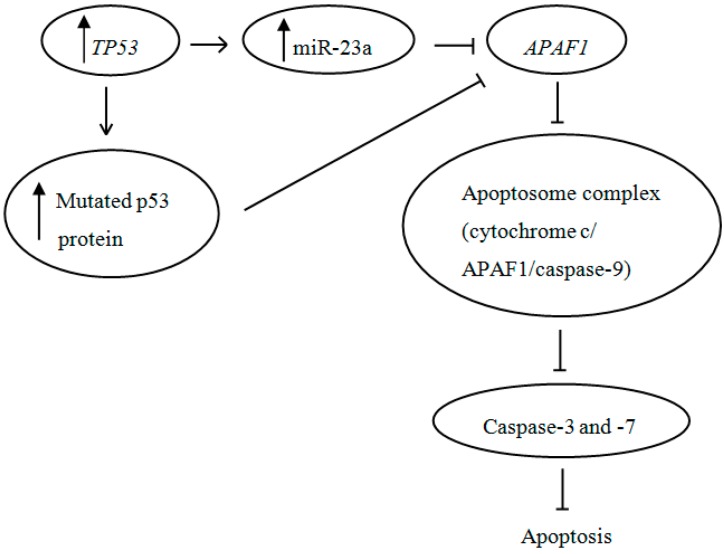
The involvement of p53 and miR-23a in the regulation of *APAF1* in CRC. The up-regulation of miR-23a and the increased synthesis of mutated p53 protein lead to a decreased synthesis of *APAF1* that interrupts the normal regulation of cell apoptosis.

Recently, Wang *et al.* have revealed that the expression of miR-23a was transcriptional activated by *TP53* gene [25]. The SW480 and SW620 cell lines used in this study have been proven to express high levels of mutated p53 proteins [26,27]. Since p53 mutation is a common genetic abnormality in CRC, the regulation of cell apoptosis via p53-dependent pathway is greatly disrupted [28]. Generally, *APAF1*, which was found to be down-regulated in our study, is an essential downstream effector of p53-mediated apoptosis [29]. *APAF1* encodes a 130 kDa protein that comprises three domains: *N*-terminal caspase recruitment domain (CARD), CED-4 like domain necessary for nucleotide binding and *C*-terminal domain containing multiple repeats of tryptophan and aspartate residues (WD repeats) responsible for protein–protein interactions [30]. In the presence of cytochrome c and dATP, APAF1 oligomerizes to form an apoptosome that is necessary to recruit downstream caspases [31]. Collectively, our findings corresponded to this notion whereby the increased expression of APAF1 following miR-23a inhibition has rescued the downstream caspase-3 and -7 activities, leading to promotion of cell apoptosis. The possible involvement of p53 and miR-23a in the regulation of *APAF1* is illustrated in Figure 8.

## 3. Experimental Section

### 3.1. Clinical Sample Collection and Preservation

A total of 70 whole blood samples and a subset of 30 paired cancer tissues with adjacent normal mucosa were collected from primary CRC patients. All participants were diagnosed and treated at the University of Malaya Medical Centre (UMMC), Malaysia, from January 2011 to January 2013. The research was performed according to the principles of the Declaration of Helsinki and has gained an approval from the UMMC Medical Ethics Committee (reference number 805.9). The recruited patients did not receive any preoperative chemotherapy and/or radiotherapy prior to surgical treatment. Tumor staging was based on the tumor-node-metastasis (TNM) system. For the control group, 32 whole blood samples that were age-, gender- and race-matched were collected from colonoscopy confirmed colonic disease-free individuals. All clinical samples were obtained with written informed consent. The clinical samples were preserved in RNA*later* solution (Ambion, Carlsbad, CA, USA).

### 3.2. Cell Culture

Human CRC cell lines, SW480 and SW620 were obtained from American Type Culture Collection (ATCC, Manassas, VA, USA). The cell lines were maintained in Dulbecco’s Modified Eagle’s Medium (DMEM) (Sigma-Aldrich, St. Louis, MO, USA) supplemented with 10% heat-inactivated fetal bovine serum (Gibco, Carlsbad, CA, USA) and 1% (*v*/*v*) penicillin/streptomycin. The cells were cultured in a 5% CO_2_-humidified incubator (MCO-17A1, Sanyo, Osaka, Japan) at 37 °C.

### 3.3. miRNA Target Prediction

miRNA target prediction was computed using miRWalk (http://www.umm.uni-heidelberg.de/apps/zmf/mirwalk). An informative prediction was assumed when a putative target was concordantly identified by five of the most common algorithms (DIANA-MicroT, miRanda, miRWalk, MirTarget2/miRDB, PicTar, RNA22, RNAhybrid and TargetScan Human) [32]. The miRNA recognition site on the 3'-UTR of *APAF1* mRNA (NM_013229.2) was then identified using TargetScan Human 6.2.

### 3.4. Total RNA Extraction

Total RNA from blood samples was extracted using Ribopure Blood RNA Isolation kit (Ambion, Carlsbad, CA, USA) while total RNA from CRC tissues and cultured cells was isolated using miRNeasy Mini kit (Qiagen, Valencia, CA, USA) according to the manufacturers’ instructions. RNA concentration was determined spectrophotometrically using NanoDrop-2000 spectrophotometer (Thermo Scientific, Wilmington, DE, USA) and the integrity was confirmed by electrophoresis on 15% polyacrylamide gel.

### 3.5. Reverse Transcription and Quantitative Real-Time PCR (RT-qPCR)

RT-qPCR for miR-23a was performed as described previously [12]. Reverse transcription for *APAF1* mRNA was performed using TaqMan reverse transcription reagents (Applied Biosystems, Carlsbad, CA, USA). qPCR was conducted on StepOnePlus real time PCR system using TaqMan fast advanced master mix and TaqMan *APAF1* gene expression assay (Assay ID: Hs00559441_m1) (Applied Biosystems, Carlsbad, CA, USA). β-actin (Assay ID: Hs99999903_m1) was chosen as the endogenous control. All assays were performed in triplicate and adhered to the protocols provided by the manufacturer. Relative expression was determined using comparative *C*_t_ (2^−∆∆*C*t^) method [33].

### 3.6. Transfection

miR-23a mimic (5'-AUCACAUUGCCAGGGAUUUCC-3'), miRNA mimic negative control (NC) (cel-miR-67: 5'-UCACAACCUCCUAGAAAGAGUAGA-3') and their inhibitors, miR-23a hairpin inhibitor and miRNA hairpin inhibitor negative control used for transfections were purchased from Dharmacon (Waltham, MA, USA). Other experimental controls include non-transfected control (NTC) and mock-transfected control. miRNA transfections for mRNA and protein analyses were performed on 48-well and 6-well plates, respectively. Briefly, SW480 and SW620 cells were seeded overnight in antibiotic-free complete medium at 1 × 10^5^ cells/well (48-well) or 1 × 10^6^ cells/well (6-well) under their normal growth conditions. After 24 h, cells were transfected using Lipofectamine 2000 transfection reagent (Invitrogen, Carlsbad, CA, USA). The RNA concentration used was 10 pmol for 48-well plate or 100 pmol for 6-well plate. Expression of *APAF1* was assayed after 48 h for mRNA analysis and 72 h for protein analysis using RT-qPCR and Western blot, respectively.

### 3.7. Total Protein Extraction and Western Blot

Total protein from CRC tissues and cultured cells was extracted using tissue protein extraction reagent (T-PER) and radio-immunoprecipitation assay (RIPA) buffer (Pierce, Rockford, IL, USA), respectively. Forty microgram (40 µg) of protein samples were then separated in 10% SDS-PAGE gel, transferred onto nitrocellulose membrane and blotted using Fast Western Blot kit, ECL substrate (Pierce, Rockford, IL, USA) based on the manufacturer’s protocol. The primary antibodies used were polyclonal anti-APAF1 (1:1000) and anti-β-actin (1:1000) (Pierce, Rockford, IL, USA). Band intensity was determined using UVP VisionWorksLS software (Upland, CA, USA).

### 3.8. Luciferase Reporter Construct

A fragment of the 3'-UTR of *APAF1* gene (NM_013229.2) containing the recognition site for miR-23a at nucleotide position 5615–5914 was constructed via geneart gene synthesis service (Invitrogen, Carlsbad, CA, USA). The corresponding site-directed mutant containing the mutated seed sequence (5'-AAUGUGAA-3' to 5'-AUUCUGCA-3') was also generated. Each customized fragment was inclusive of the HindIII and SpeI restriction sites (312 bp). The wild type and mutated *APAF1* 3'-UTR fragments were then cloned into pMIR-REPORT firefly luciferase vector (Ambion, Carlsbad, CA, USA) at the HindIII and SpeI sites and designated as Luc-APAF1-wt and Luc-APAF1-mt, respectively. A luciferase vector without *APAF1* 3'-UTR (Luc-APAF1-ctl) was used as experimental control. All constructs were confirmed by DNA sequencing.

### 3.9. Luciferase Assay

The co-transfection procedure for miRNAs and luciferase reporter constructs was performed using Lipofectamine 2000 reagent (Invitrogen, Carlsbad, CA, USA). SW480 cells were seeded overnight in antibiotic-free complete medium at 2 × 10^4^ cells/well on a 96-well white plate. The cells were transfected with 5 pmol of miR-23a mimic, miR-23a inhibitor or negative controls for 3 h followed by 100 ng of Luc-APAF1-ctl, Luc-APAF1-wt or Luc-APAF1-mt together with 10 ng of *Renilla* luciferase vector (pRL-SV40) for another 3 h. Luciferase activities were measured after 48 h using Dual-Glo Luciferase assay (Promega, Madison, WI, USA). Transfection efficiencies were normalized by dividing the firefly luciferase signal with that of *Renilla* luciferase.

### 3.10. Cell Viability Assay

The cell viability of SW480 and SW620 cells post transfections (24, 48 and 72 h) was measured using MTT Cell Proliferation kit (Oz Biosciences, Marseille, France). Briefly, 2 × 10^4^ cells were seeded overnight in 96-well plate and transfection was carried out accordingly using Lipofectamine 2000 reagent (Invitrogen, Carlsbad, CA, USA). At the indicated time points, culture medium was removed and 100 µL of 1× MTT working solution was added. The plate was left at 37 °C for 4 h. At the end of the incubation, 100 μL of solubilization solution was added. The absorbance was measured at a wavelength of 570 nm and a reference wavelength at 650 nm.

### 3.11. Apoptosis Assay and Image-Based Cytometry

The apoptosis assay was performed using Annexin V-FITC apoptosis detection kit (BioVision, Milpitas, CA, USA). miRNA transfection was conducted accordingly using 6-well plate. After 48 h of transfection, 2 × 10^5^ cells were harvested and resuspended in 1× binding buffer for staining with annexin V-FITC and propidium iodide (PI). The sample (25 µL) was loaded onto a Tali cellular analysis slide (Invitrogen, Carlsbad, CA, USA). Quantitative analyses of live (annexin V-negative/PI negative), dead (annexin V positive/PI positive) and apoptotic (annexin-V positive/PI negative) cell populations were evaluated using Tali image-based cytometer (Invitrogen, Carlsbad, CA, USA). An unstained sample was initially analyzed and set as the background in order to determine the fluorescent thresholds. Each sample was then gated according to the initial thresholds.

### 3.12. Caspase-Glo 3/7 Assay

Caspase-Glo 3/7 assay (Promega, Madison, WI, USA) was used to measure the activation of caspase-3 and -7 activities. Cells (2 × 10^4^ cells/well) were seeded overnight in 96-well white plate. Transfected cells were incubated in the CO_2_ incubator at 37 °C for 48 h. At the end of the incubation, 100 µL of Caspase-Glo 3/7 reagent was added and the plate was kept at room temperature for 3 h prior to luminescence detection.

### 3.13. Statistical Analysis

Data were expressed as mean ± standard error of mean (SEM). Statistical significance was determined by paired or unpaired *t*-test and a two-tailed *p*-value of <0.05 was considered to be statistically significant. Correlation analysis was determined by Pearson’s test. Statistical analysis was performed using SPSS version 16.0 software (IBM, Armonk, NY, USA).

## 4. Conclusions

In conclusion, miR-23a up-regulation was found to be coupled with *APAF1* down-regulation in CRC tissue samples. miR-23a was demonstrated to possess apoptosis resistance function in CRC by targeting *APAF1*. Our work highlights the importance of further investigation using animal models and randomized clinical studies to further elucidate the regulative mechanism underlying the miR-23a/*APAF1* interaction, as well as other miRNAs that might be involved in the apoptotic regulation of CRC.

## Supplementary Files

Supplementary File 1

## References

[1] Bray F., Jemal A., Grey N., Ferlay J., Forman D. (2012). Global cancer transitions according to the human development index (2008–2030): A population-based study. Lancet Oncol..

[2] Bartel D.P. (2004). MicroRNAs: Genomics, biogenesis, mechanism, and function. Cell.

[3] Chan S.P., Slack F.J. (2006). MicroRNA-mediated silencing inside p-bodies. RNA Biol..

[4] Liu X., Fortin K., Mourelatos Z. (2008). MicroRNAs: Biogenesis and molecular functions. Brain Pathol..

[5] Wu L., Fan J., Belasco J.G. (2006). MicroRNAs direct rapid deadenylation of mRNA. Proc. Natl. Acad. Sci. USA.

[6] Bartel D.P. (2009). MicroRNAs: Target recognition and regulatory functions. Cell.

[7] Lynam-Lennon N., Maher S.G., Reynolds J.V. (2009). The roles of microRNA in cancer and apoptosis. Biol. Rev. Camb. Philos. Soc..

[8] Tsuchida A., Ohno S., Wu W., Borjigin N., Fujita K., Aoki T., Ueda S., Takanashi M., Kuroda M. (2011). miR-92 is a key oncogenic component of the miR-17-92 cluster in colon cancer. Cancer Sci..

[9] Asangani I.A., Rasheed S.A., Nikolova D.A., Leupold J.H., Colburn N.H., Post S., Allgayer H. (2008). MicroRNA-21 (miR-21) post-transcriptionally downregulates tumor suppressor Pdcd4 and stimulates invasion, intravasation and metastasis in colorectal cancer. Oncogene.

[10] Yamamichi N., Shimomura R., Inada K., Sakurai K., Haraguchi T., Ozaki Y., Fujita S., Mizutani T., Furukawa C., Fujishiro M. (2009). Locked nucleic acid in situ hybridization analysis of miR-21 expression during colorectal cancer development. Clin. Cancer Res..

[11] Chang T.C., Wentzel E.A., Kent O.A., Ramachandran K., Mullendore M., Lee K.H., Feldmann G., Yamakuchi M., Ferlito M., Lowenstein C.J. (2007). Transactivation of miR-34a by p53 broadly influences gene expression and promotes apoptosis. Mol. Cell.

[12] Yong F.L., Law C.W., Wang C.W. (2013). Potentiality of a triple microRNA classifier: miR-193a-3p, miR-23a and miR-338-5p for early detection of colorectal cancer. BMC Cancer.

[13] Krek A., Grün D., Poy M.N., Wolf R., Rosenberg L., Epstein E.J., MacMenamin P., da Piedade I., Gunsalus K.C., Stoffel M. (2005). Combinatorial microRNA target predictions. Nat. Genet..

[14] Leibovitz A., Stinson J.C., McCombs W.B.I., McCoy C.E., Mazur K.C., Mabry N.D. (1976). Classification of human colorectal adenocarcinoma cell lines. Cancer Res..

[15] Doench J.G., Sharp P.A. (2004). Specificity of microRNA target selection in translational repression. Genes Dev..

[16] Huerta S., Heinzerling J.H., Anguiano-Hernandez Y.M., Huerta-Yepez S., Lin J., Chen D., Bonavida B., Livingston E.H. (2007). Modification of gene products involved in resistance to apoptosis in metastatic colon cancer cells: Roles of Fas, Apaf-1, NFkappaB, IAPs, Smac/DIABLO, and AIF. J. Surg. Res..

[17] Hewitt R.E., McMarlin A., Kleiner D., Wersto R., Martin P., Tsokos M., Stamp G.W., Stetler-Stevenson W.G. (2000). Validation of a model of colon cancer progression. J. Pathol..

[18] Li B., Sun M., Gao F., Liu W., Yang Y., Liu H., Cheng Y., Liu C., Cai J. (2013). Up-regulated expression of miR-23a/b targeted the pro-apoptotic Fas in radiation-induced thymic lymphoma. Cell Physiol. Biochem..

[19] Guo Z., Zhou B., Liu W., Xu Y., Wu D., Yin Z., Permatasari F., Luo D. (2013). miR-23a regulates DNA damage repair and apoptosis in UVB-irradiated HaCaT cells. J. Dermatol. Sci..

[20] Paik S.S., Jang K.S., Song Y.S., Jang S.H., Min K.W., Han H.X., Na W., Lee K.H., Choi D., Jang S.J. (2007). Reduced expression of Apaf-1 in colorectal adenocarcinoma correlates with tumor progression and aggressive phenotype. Ann. Surg. Oncol..

[21] Zlobec I., Minoo P., Baker K., Haegert D., Khetani K., Tornillo L., Terracciano L., Jass J.R., Lugli A. (2007). Loss of APAF-1 expression is associated with tumour progression and adverse prognosis in colorectal cancer. Eur. J. Cancer.

[22] Luo X., Burwinkel B., Tao S., Brenner H. (2011). MicroRNA signatures: Novel biomarker for colorectal cancer?. Cancer Epidemiol. Biomark. Prev..

[23] Volinia S., Calin G.A., Liu C.G., Ambs S., Cimmino A., Petrocca F., Visone R., Lorio M., Roldo C., Ferracin M. (2006). A microRNA expression signature of human solid tumors defines cancer gene targets. Proc. Natl. Acad. Sci. USA.

[24] Meltzer P.S. (2005). Cancer genomics: Small RNAs with big impacts. Nature.

[25] Wang N., Zhu M., Tsao S.W., Man K., Zhang Z., Feng Y. (2013). miR-23a-mediated inhibition of topoisomerase 1 expression potentiates cell response to etoposide in human hepatocellular carcinoma. Mol. Cancer.

[26] Rodrigues N.R., Rowan A., Smith M.E., Kerr I.B., Bodmer W.F., Gannon J.V., Lane D.P. (1990). p53 mutations in colorectal cancer. Proc. Natl. Acad. Sci. USA.

[27] Violette S., Poulain L., Dussaulx E., Pepin D., Faussat A.M., Chambaz J., Lacorte J.M., Staedel C., Lesuffleur T. (2002). Resistance of colon cancer cells to long-term 5-fluorouracil exposure is correlated to the relative level of bcl-2 and bcl-xl in addition to bax and p53 status. Int. J. Cancer.

[28] Lacopetta B. (2003). TP53 mutation in colorectal cancer. Hum. Mutat..

[29] Robles A.I., Bemmels N.A., Foraker A.B., Harris C.C. (2001). APAF-1 is a transcriptional target of p53 in DNA damage-induced apoptosis. Cancer Res..

[30] Campioni M., Santini D., Tonini G., Murace R., Dragonetti E., Spugnini E.P., Baldi A. (2005). Role of Apaf-1, a key regulator of apoptosis, in melanoma progression and chemoresistance. Exp. Dermatol..

[31] Cain K., Bratton S.B., Cohen G.M. (2002). The apaf-1 apoptosome: A large caspase-activating complex. Biochimie.

[32] Dweep H., Sticht C., Pandey P., Gretz N. (2011). miRWalk-database: Prediction of possible miRNA binding sites by “walking” the genes of 3 genomes. J. Biomed. Inform..

[33] Yuan J.S., Reed A., Chen F., Stewart C.N. (2006). Statistical analysis of real-time PCR data. BMC. Bioinform..

