# Supplementary material for: The Involvement of miR-23a/APAF1 Regulation Axis in Colorectal Cancer
Source: Int J Mol Sci. 2014 Jul 2;15(7):11713–29. doi: 10.3390/ijms150711713 (PMC4139809; doi:10.3390/ijms150711713)
Supplement: Supplementary File 1 [file ijms-15-11713-s001.pdf]

Supplementary Information

Table S1. Expression levels of miR-23a and *APAF1* in clinical carcinogenesis of colorectal cancer (CRC) samples. (**A**) Endogenous expressions of miR-23a and *APAF1* mRNA in the blood samples obtained from 32 healthy controls (CTL) and 70 CRC patients (C); assayed using RT-qPCR and expressed as fold change ($2^{-\Delta\Delta Ct}$) value with respect to the healthy controls; and (**B**) Endogenous expressions of miR-23a, *APAF1* mRNA and APAF1 protein in the subset of 30 paired cancer tissues. Tissue miR-23a and *APAF1* mRNA levels were determined by RT-qPCR while tissue APAF1 protein expression was detected by Western blot. Expression value was calculated as fold change of cancer tissue versus the adjacent normal mucosa.

Table S1. (**A**).

Blood Sample	Fold Change		Blood Sample	Fold Change	
	miR-23a	*APAF1* mRNA		miR-23a	*APAF1* mRNA
CTL_WB	1.2980	1.0336	C_KX	5.1983	0.3260
CTL_WC	2.1623	0.6431	C_KY	0.5935	0.5320
CTL_WD	2.7579	0.6115	C_KZ	2.0115	0.2042
CTL_WF	2.3725	0.8409	C_LA	0.5299	0.4724
CTL_XF	0.5650	1.8544	C_LC	3.9284	0.4523
CTL_XL	0.7041	2.4242	C_LD	1.1560	0.3610
CTL_XM	4.3584	0.4337	C_LE	4.1912	0.4696
CTL_XU	0.9979	2.1795	C_LF	3.4732	0.1846
CTL_XZ	0.6516	0.9474	C_LG	0.5006	1.4162
CTL_YA	0.7401	1.3008	C_LI	2.0944	0.5052
CTL_YC	1.2297	1.3128	C_LJ	1.1709	0.5610
CTL_YD	2.2761	0.9059	C_LK	1.0853	0.5820
CTL_YF	0.9529	2.4128	C_LM	0.9533	0.2313
CTL_YH	1.3371	1.7461	C_LN	0.3992	0.3053
CTL_YJ	0.5298	0.7091	C_LO	1.1179	1.0678
CTL_YL	0.7726	1.2841	C_LP	0.0724	0.2916
CTL_YN	2.6184	1.3244	C_LQ	1.4897	2.5796
CTL_YO	2.4846	0.5803	C_LR	0.2032	0.1669
CTL_YQ	1.0988	2.3799	C_LS	1.2425	1.4301
CTL_YW	4.5931	1.8306	C_LU	2.6450	0.2668
CTL_ZB	0.1561	1.2619	C_LX	2.0178	1.3228
CTL_ZF	0.1544	2.5886	C_LY	2.9558	1.1050
CTL_ZJ	0.1636	1.9763	C_LZ	1.1938	1.7919
CTL_ZM	0.6988	1.3223	C_MA	1.1491	0.4676
CTL_ZN	1.2166	2.9708	C_MB	3.0863	0.4327
CTL_ZP	2.9786	0.3963	C_MC	0.7103	1.4348
CTL_ZQ	0.1874	0.2099	C_MD	1.6959	0.7204
CTL_ZR	0.1758	0.1302	C_MG	1.0279	1.7903

Table S1. (**A**). *Cont.*

Blood Sample	Fold Change		Blood Sample	Fold Change	
	**miR-23a**	***APAF1* mRNA**		**miR-23a**	***APAF1* mRNA**
CTL_ZT	0.7534	0.1883	C_MI	2.2409	0.2308
CTL_ZW	1.0035	0.4654	C_MK	1.2518	0.1457
CTL_ZY	1.8724	1.5038	C_ML	2.2431	0.3427
CTL_ZZ	2.9238	1.0102	C_MN	2.6407	0.1910
C_C	10.0868	1.2307	C_MO	0.7173	1.1146
C_F	0.7284	1.2467	C_MP	2.7526	1.2497
C_G	6.7512	0.3446	C_MS	0.7668	0.2584
C_H	1.0948	0.4548	C_MV	1.7642	1.3794
C_KA	4.4825	0.9426	C_MW	2.7149	0.2476
C_KB	1.6411	0.9520	C_MX	2.5130	0.3123
C_KD	1.5969	0.4207	C_MY	2.8983	0.9169
C_KF	0.8814	1.3852	C_MZ	1.2831	0.3894
C_KG	0.8886	0.1956	C_NA	0.7897	0.1392
C_KH	1.1223	1.0171	C_ND	2.2929	1.5791
C_KI	0.9392	1.4141	C_NG	1.7315	1.2542
C_KJ	0.8325	1.3964	C_NH	1.7607	1.7045
C_KK	0.7216	0.5864	C_NI	2.0009	0.8834
C_KM	2.2030	0.3590	C_NL	2.8009	1.2504
C_KN	4.5832	0.6276	C_NN	1.4899	0.8348
C_KR	0.9345	0.3744	C_NQ	1.7284	0.2657
C_KS	1.1212	1.0523	C_NR	0.9127	0.1450
C_KT	2.6839	0.8877	C_NS	0.8104	1.3975
C_KU	0.7299	0.2022	C_NV	1.6145	1.4826

Table S1. (B).

**Paired Tissue Sample**	**Fold Change**		
	**miR-23a**	***APAF1* mRNA**	**APAF1 Protein**
C_KB	0.8183	0.3013	0.4469
C_KF	0.5263	0.3939	0.7943
C_KG	1.0981	0.3946	0.9720
C_KI	0.6040	0.7304	1.2070
C_KN	3.4987	0.1597	0.8361
C_KS	1.7579	0.1089	0.6136
C_KU	0.7696	0.0221	1.0867
C_KX	6.6793	0.0800	0.6592
C_KY	0.5172	0.4202	0.7827
C_LC	2.0456	0.1775	0.3421
C_LE	5.6637	0.1506	0.4178
C_LF	5.0208	0.1050	0.6098
C_LJ	2.2800	0.1404	0.8293
C_LK	2.5638	0.4111	0.8709
C_LM	2.4835	0.0774	0.4970
C_LP	0.1329	0.5655	0.8649
C_LR	0.7905	0.1955	1.1586
C_LX	1.7387	0.1741	0.6734
C_LY	4.7860	0.1548	0.5479
C_ML	3.9549	0.0482	0.8409
C_MN	3.0303	0.0437	0.5981
C_MS	0.3240	0.2913	1.1646
C_MV	1.1640	0.1930	0.3855
C_MY	4.0591	0.0959	0.5341
C_NA	0.1988	0.2330	1.1435
C_NH	1.8119	0.2538	0.7696
C_NI	2.1900	0.1603	0.4717
C_NQ	1.4088	0.1835	0.9567
C_NR	0.9086	0.2163	0.5861
C_NV	3.8037	0.7431	0.8116
